# Automated Age Estimation from OPG Images and Patient Records Using Deep Feature Extraction and Modified Genetic–Random Forest

**DOI:** 10.3390/diagnostics15030314

**Published:** 2025-01-29

**Authors:** Gulfem Ozlu Ucan, Omar Abboosh Hussein Gwassi, Burak Kerem Apaydin, Bahadir Ucan

**Affiliations:** 1 Department of Oral and Maxillofacial Radiology, Faculty of Dentistry, Istanbul Gelisim University, Istanbul 34310, Turkey; 2 Electrical and Computer Engineering, School of Engineering and Natural Sciences, Altinbas University, Istanbul 34217, Turkey; 213720043@ogr.altinbas.edu.tr; 3 Department of Oral and Maxillofacial Radiology, Faculty of Dentistry, Pamukkale University, Denizli 20160, Turkey; drkeremapaydin@gmail.com; 4 Department of Communication and Design, Yildiz Technical University, Istanbul 34220, Turkey; bucan@yildiz.edu.tr

**Keywords:** age estimation, dental age estimation, forensic odontology, deep learning, machine learning, forensics, panoramic radiograph

## Abstract

**Background/Objectives:** Dental age estimation is a vital component of forensic science, helping to determine the identity and actual age of an individual. However, its effectiveness is challenged by methodological variability and biological differences between individuals. Therefore, to overcome the drawbacks such as the dependence on manual measurements, requiring a lot of time and effort, and the difficulty of routine clinical application due to large sample sizes, we aimed to automatically estimate tooth age from panoramic radiographs (OPGs) using artificial intelligence (AI) algorithms. **Methods:** Two-Dimensional Deep Convolutional Neural Network (2D-DCNN) and One-Dimensional Deep Convolutional Neural Network (1D-DCNN) techniques were used to extract features from panoramic radiographs and patient records. To perform age estimation using feature information, Genetic algorithm (GA) and Random Forest algorithm (RF) were modified, combined, and defined as Modified Genetic–Random Forest Algorithm (MG-RF). The performance of the system used in our study was analyzed based on the MSE, MAE, RMSE, and R^2^ values calculated during the implementation of the code. **Results:** As a result of the applied algorithms, the MSE value was 0.00027, MAE value was 0.0079, RMSE was 0.0888, and R^2^ score was 0.999. **Conclusions:** The findings of our study indicate that the AI-based system employed herein is an effective tool for age detection. Consequently, we propose that this technology could be utilized in forensic sciences in the future.

## 1. Introduction

Identification of individuals represents a crucial area within the discipline of forensic sciences [1,2]. The age of the individual in question carries significant weight in the process of identification [3,4]. In instances of mass disasters, organized crime, and abuse cases, it is imperative to ascertain the age of suspects and victims to facilitate their identification [5]. In the extant literature, the estimation of age in human history and forensic sciences has consistently been highlighted as a research topic, with the objective of developing reliable age estimation techniques for both living and deceased individuals [4].

The term chronological age is used to describe the time that has elapsed since an individual’s date of birth. This is calculated by determining the dates of both the individual’s birth and death. In the absence of available data regarding the dates of birth and death, the age of an individual can be estimated by examining the biological age of the individual in question. The assessment of biological age is based on the physical development stages of the individual or the changes that occur with aging. This assessment includes the development of various systems such as height, weight, hair, skin, eyes, teeth, bones, and secondary sex characteristics [6,7,8].

Dental tissues are the most durable part of the skeleton [9]. They are resistant to extreme conditions and are preserved longer than bone [10]. For this reason, teeth are often used in forensic science to estimate age [11]. Even in cases of major disasters, when the victim’s body is so mutilated that it cannot be visually identified, the remains of skull bones, jaw bones, and teeth have proven to be the most valuable source of identification [4,12]. Radiographic estimation of age and sex from radiographs of the jaw bones is considered more feasible because it is a simple and less destructive method that can be applied to both deceased and living cases [4,13].

A variety of methods, including morphological, metric, radio morphological, radio metric, histological, and biochemical approaches, can be utilized for age estimation in dental tissues [2,14,15]. However, further research is necessary to ascertain the applicability and reliability of these methods across diverse populations [11]. The classic age estimation methods rely on manual measurements and observer subjectivity, which are time-consuming and prone to observer bias, potentially increasing the workload of forensic experts and introducing subjectivity into the estimation process [16,17].

AI is defined as the capacity of a machine to imitate the cognitive processes and behaviors observed in humans, enabling the completion of tasks that would otherwise require human input. In recent years, the rapid development of AI has facilitated numerous technological advancements that have enhanced the quality of life for many individuals. Furthermore, AI has advanced rapidly in numerous medical domains, garnering significant interest in recent years, particularly within the radiology community. To date, the implementation of AI in dental radiography has demonstrated considerable potential for a multitude of applications. At this point, it can facilitate critical support for clinicians, novice physicians, and students in the decision making process [18].

In recent years, studies based on AI have been conducted with the objective of automating tasks in dentistry. These include caries diagnosis and classification [19,20,21,22,23,24,25,26,27,28], automatic diagnosis of dental diseases and conditions of the teeth [29], diagnostic evaluation and segmentation in periapical radiographs [30], periodontal bone loss [31,32], implant planning [33], detection and classification of periapical pathologies [34], cephalometric analysis and automatic detection of anatomical landmarks [35,36], detection of impacted mandibular third molars, and [37] to detecting and classifying impacted maxillary supernumerary teeth [38,39,40,41,42]. Additionally, research has aimed to detect vertical root fractures [43,44], classify osteoporosis [45,46,47], improve image quality [48,49,50], identify and classify odontogenic tumors and cysts [51,52], diagnose maxillary sinus pathologies [53,54,55], identify and classify lymph node metastases [56,57], diagnose patients with Sjogren syndrome [58], and detect oral cancer lesions [59].

AI also offers significant potential in the field of forensic sciences for data analysis and ensuring the proper administration of justice [60]. These models have the potential to be promising tools when identifying victims of mass disasters and as an additional aid in medico-legal situations. In the literature, AI-based models have been reported to show similar accuracy and precision as trained forensic scientists. It has been stated that these models can be promising tools when identifying victims of mass disasters and as an additional aid in medico-legal situations [61].

The advent of deep learning represents a significant milestone in the evolution of AI algorithms. Deep learning is a group of algorithms based on the structure of artificial neural networks and defined as a sub-branch of machine learning. Deep learning algorithms offer significant advantages in processing complex datasets and making sense of problems. These algorithms simplify complex problems by classifying datasets in a hierarchical manner through the use of multilayer artificial neural networks. A typical deep learning system consists of an input layer representing the dataset of the problem, multiple hidden layers, and an output layer. Hidden layers contain links that help to understand the relationships in the dataset. This structure provides more effective results in solving complex problems than classical AI algorithms [62].

Existing classic age estimation methods have been critiqued in the literature for several shortcomings. Observer subjectivity has been identified as a potential source of error in age estimation. Furthermore, manual measurement-based methods are time-consuming and laborious. Additionally, the feasibility of implementing these methods in routine clinical practice has been questioned due to the limited sample sizes typically used. Considering the considerations, the present research endeavors to employ DM-based methodologies for the processing of panoramic radiograph (OPG) images and patient records, with the objective of estimating the age of individuals with the highest estimation rate.

This study utilized the Deep 2D CNN for feature extraction from OPG images and Deep 1D CNN for feature extraction from patient records, hence establishing a dual-feature extraction process resulting in a more robust and comprehensive representation of patient data. Following that, employing a concatenate strategy to merge features from these two approaches enhanced the predictive performance of age estimation models. Furthermore, the study proposes an MG-RF regressor that combines the optimization ability of the GA with RF algorithm. It can minimize overfitting, enhance the detection rate, and utilize genetic indications via chromosomes, affording numerous optimal solutions. The study implemented this innovative methodology specifically for pediatric age estimation, a vital task in both medical and forensic fields. Regarding these advantages, the present work considers this AI-based methodology for age estimation.

The primary objectives of this study were as follows:

- To extract pertinent features from OPG images and patient records, employing innovative approach that combines Deep 2D CNN with a Deep 1D CNN. The extracted features were then concatenated to improve the accuracy of age estimation;
- To achieve the highest coefficient of determination (R^2^) for age estimation by leveraging an MG-RF regressor;
- To evaluate the efficiency of the proposed methodology with respect to standard deviation (SD), mean absolute error (MAE), mean square error (MSE), root mean square error (RMSE), and R^2^.

## 2. Related Work

In recent years, AI-based methodologies have been developed with the objective of overcoming these limitations and automating the age estimation process. These approaches yield consistent and reproducible results and facilitate reduced processing times. These approaches are deemed capable of detecting attributes that are not recognizable to human observers and are not feasible for manual computation. Moreover, the substantial benefits in identifying intricate relationships between features facilitate more accurate predictions with diminished error rates [17,63,64]. AI-based approaches in the literature are generally categorized as machine learning, deep learning, or a combination of these two methodologies [17]. The objectives of these studies include numerical age regression [16,17,65,66,67,68,69,70,71], staging of teeth [72,73,74], classification of age groups [75,76], and legal age classification [77].

Galibourg et al. [66] conducted a study with a machine learning approach for numerical age regression. The researchers employed ten machine learning methods, including Random Forest (RF), Support Vector Machine (SVM), Decision Tree (DT), Bayesian Ridge Regression (BRR), K-Nearest Neighbors (KNN), AdaBoost (ADAB), Polynomial Regression (POLYREG), Multi-Layer Perceptron (MLP), Stacking (STACK), and Voting (VOTE). The researchers reported that age estimation using machine learning methods yielded superior results compared to manual methods based on radiographic dental staging from childhood to early adulthood.

In addition, one study employed Scaled-YOLOv4 to detect dental germs with 8023 panoramic radiographs as training data, achieving MAP, EfficientNetV2, and M classifiers for the developmental stages of detected dental germs using 18,485 single-root and 16,313 multi-root images. This approach yielded superior outcomes for multi-root classifications. The MAE between automatic and manual dental age calculations using different methods was 0.274 for single selection, 0.261 for weighted average, and 0.396 for the expected value, with the weighted average demonstrating the most optimal performance [78].

Similarly, Tao et al. [67] employed machine learning for numerical age regression. The researchers employed an MLP for this purpose. It was demonstrated that the proposed system exhibited superior performance compared to the reference manual methods across all performance metrics.

A DL model, designated as DentAge, was developed for the automated estimation of age using panoramic dental X-ray images. This model was trained on a dataset comprising 21,007 images from a private dental center in Slovenia, encompassing subjects ranging in age from 4 to 97 years. The model attained an MAE of 3.12 years across the test dataset, thereby substantiating its efficacy in age estimation under varied dental conditions [79].

Shen et al. [68] employed machine learning for numerical age regression, utilizing RF, SVM, and linear regression (LR). The findings of the study indicated that age estimation accuracy was superior in machine learning methods compared to traditional methods.

De Tobel [72] employed a deep learning approach for staging teeth, with transfer learning (Alex-Net) demonstrating the most effective performance for staging.

Boedi et al. [73] employed the Dense Net machine learning algorithm. The objective of the present study was to ascertain and validate the impact of lower third molar segmentations on automatic tooth development staging. The study’s findings led to the conclusion that full tooth segmentation and DenseNet CNN optimization facilitate the accurate allocation of dental stages.

Banar et al. [74] employed a CNN that was similar in structure to the You Only Look Once (YOLO) algorithm, in conjunction with the U-Net CNN and the DenseNet201 CNN. The objective of this study is to automate the staging process in its entirety, utilizing CNN at each stage of the procedure. The results demonstrate that the proposed fully automated approach yields promising outcomes in comparison to manual staging.

In their study, Kim et al. [75] employed a deep learning approach, ResNet 152, for the classification of age groups. The accuracy of the tooth-wise prediction was found to be between 89.05 and 90.27 percent. The performance accuracy was primarily assessed through the use of the majority voting system and area under the curve (AUC) scores. The AUC scores ranged from 0.94 to 0.98 for all age groups, indicating a superior capability.

Dong et al. [76] developed a methodology for identifying tooth maturity stages in fully permanent dentition. This methodology features a YOLOv3-based tooth localization model for detecting and numbering teeth, a symmetric ordinal staging network (SOS-Net) that enhances feature representation while minimizing parameters, and an auxiliary regression branch with adjacent stage-aware (ASA) loss to reduce misclassification. The efficacy of this methodology was evaluated using a private OPG dataset comprising subjects between the ages of 3 and 14 years. The experimental results obtained demonstrated enhanced F1-scores, thereby surpassing contemporary state-of-the-art methodologies in terms of maturity staging and age estimation.

Guo et al. [77] employed the SE-ResNet101 model to ascertain the legal age groups. It was reported that end-to-end CNN models demonstrated superior performance, with accuracy rates of 92.5%, 91.3%, and 91.8% for age thresholds of 14, 16, and 18 years, respectively. To-end CNN models demonstrated superior performance, with accuracy rates of 95.9%, 95.4%, and 95.4% for age thresholds of 14, 16, and 18 years, respectively.

Čular et al. [69] employed a combination of Deep Learning Active Shape Model (ASM), Active Appearance Model (AAM), and Radial Basis Network algorithms for the purpose of numerical age regression. In this study, the researchers proposed a semi-automated system based on deep learning techniques to predict tooth age by analyzing the mandibular right third molar tooth on OPGs.

De Back et al. [70] applied deep learning and Bayesian convolutional neural networks methods for numerical age regression. The system achieved a concordance correlation coefficient of ccc = 0.91 in the validation set.

A three-step framework for estimating dental age in children aged 3 to 15 was also developed. This framework includes the following elements: the employment of a YOLOv3 network for tooth localization and numbering, the achievement of a mean average precision, and the establishment of a novel SOS-Net for accurate tooth development staging based on a modified Demirjian method. The result of these efforts was an average accuracy of 82.97% for full dentition. In addition, a dental age assessment was conducted through a single-group meta-analysis, yielding an MAE of 0.72 years when excluding third molars [80].

Wallraff et al. [71] employed the ResNet18 algorithm. In this study, a supervised regression-based deep learning method for automatic age estimation of adolescents aged 11 to 20 years was proposed as a means of reducing the estimation error. In an initial investigation, the proposed methodology demonstrated a mean absolute error (MAE) of 1.08 years and an error rate (ER) of 17.52% on the test dataset, exhibiting superior performance compared to the predictions of dental experts.

In their study, Vila-Blanco et al. [65] employed Rotated R-CNN algorithms to propose a novel, fully automatic methodology for age and gender estimation. The method initially employs a modified CNN to detect teeth and extract oriented bounding boxes for each tooth. These boxes are then fed into a second CNN module, designed to produce probability distributions of age and gender per tooth. Finally, an uncertainty-sensitive approach is used to aggregate these estimated distributions, resulting in an improvement in the absolute error rate.

## 3. Materials and Methods

This study was approved by the Pamukkale University Non-Interventional Clinical Research Ethics Committee (E-60116787-020-202083/26 April 2022). The study was conducted in accordance with the principles set forth in the Declaration of Helsinki.

**Figure 1:**
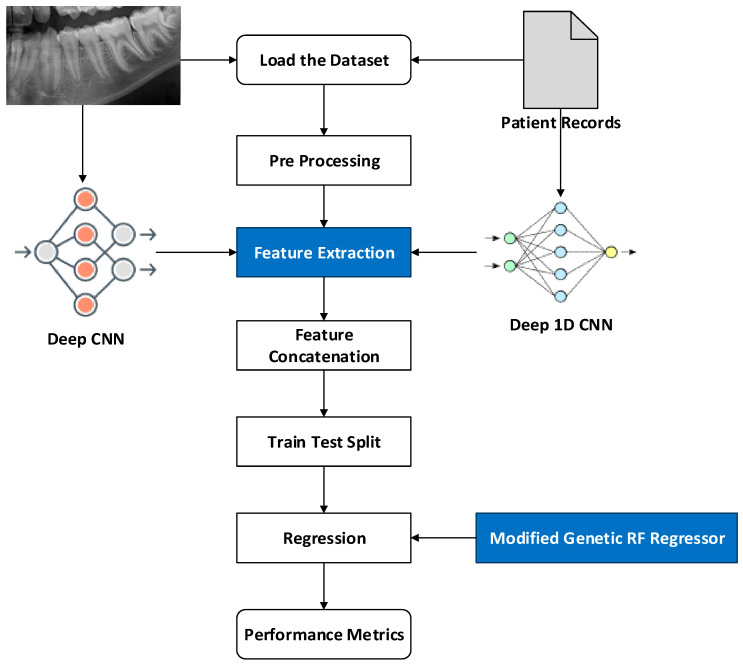
Overall view of the proposed system. Feature extraction is a key step in machine learning and data analysis, that is colored to emphasize its role on transferring data in between Deep CNN and Deep 1D CNN. Modified random forest regression is a type of supervised learning algorithm that employs an ensemble approach to tackle regression tasks.

In this study, feature extraction is individually performed using the proposed Deep 2D CNN and Deep 1D CNN. In addition, MG-RF is proposed for estimating the age. The proposed methodologies estimate age through the sequence of processes shown in Figure 1. Initially, the dataset was loaded. Subsequently, pre-processing was undertaken to make the data easier to interpret and use. This process also assisted in eliminating the duplicates or inconsistencies in the data that could otherwise negatively impact the prediction rate of the model. Preprocessing the data also assisted in ensuring that there were no missing or incorrect data due to bugs or human error. Following this, feature extraction was performed for eliminating the redundant data. Deep 2D CNN was used to extract the features from OPG images. Simultaneously, Deep ID CNN was utilized to extract features from patient records. These features were individually extracted and then concatenated to attain suitably efficient features for better age estimation using MG-RF. Finally, performance metrics were utilized to validate the effectiveness of the proposed system in comparison to existing methods.

### 3.1. Dataset Description

The present study included all systemically healthy patients between the ages of 6 and 15 years who had undergone an OPG in the correct position, yielding an OPG free of artifacts and distortions that was clearly evaluable, and who did not have any dental deficiencies and dental restorations. The archive of the Department of Oral, Dental and Maxillofacial Radiology, Faculty of Dentistry, Pamukkale University, was the source of the data. The dataset was created between 1 March 2020 and 1 March 2022. The patients’ chronological age was calculated by subtracting the date of birth from the date of the OPGs.

OPGs were obtained with a digital orthopantomograph (OP200D; Instrumentarium Company, Imaging Unit, Tuusula, Finland) with exposure values between 66 kVp, 2.5 mA, and 13.4 s and 60 kVp, 6.3 mA, and 14.1 s. The OPGs were subsequently evaluated using AI algorithms.

The dataset consists of OPGs and corresponding patient records intended for research purposes and utilized in real-time applications. It includes patients within a specific age range, as indicated by the average ages of males and females, highlighting a focus on a particular developmental stage. Both genders are represented, and images are selected based on clinical relevance, such as the presence of dental conditions or anomalies, ensuring that only high-quality OPGs with sufficient diagnostic value are included. The distribution of images in the dataset, which consists of orthopantomogram (OPG) images and patient records, is characterized by the features of 275 male and 346 female patients, suggesting a minimum of 621 images if one image per patient is assumed. Out of 275 male patients, the average age is 10.94, while out of 346 female, average age is 11.1.

### 3.2. Preprocessing

The preprocessing pipeline for patient data comprises several essential steps to ensure high-quality input for DL models. Initially, data cleaning is performed to eliminate any missing information, followed by the application of a normalization approach, such as min–max scaling for OPG images and Z-score method for patient records (numeric data). The min–max technique is applied to adjust the pixel intensity values of images, transforming the data into a specified range, generally as [0, 1]. The Z-score method is employed to convert numerical data into a standard normal distribution, thereby facilitating more efficient analysis. Consequently, this step enhances the quality of the input image, leading to an improved prediction accuracy.

### 3.3. Feature Extraction: Deep Two-Dimensional Convolution Neural Network and Deep One-Dimensional Convolution Neural Network

Recently, DL has gained paramount significance in the medical domain, as it possesses the capability for handling huge data. Thus, this study employed two Deep CNN models to perform feature extraction. The Deep 2D CNN model was applied to extract features from OPG images, while Deep 1D CNN was utilized for extracting features from the patient records. After extracting individual features, these were concatenated and fed into the trained model for age estimation.

#### 3.3.1. Deep 2D CNN: Deep Two-Dimensional Convolutional Neural Network

**Figure 2:**
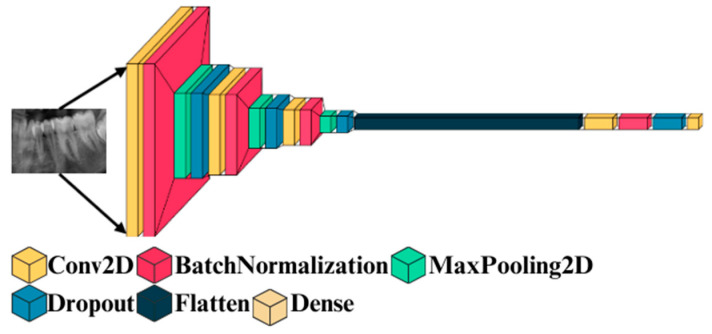
Overall architecture of Deep 2D CNN.

Deep 2D CNN is a standard CNN and has obtained use in extensive application areas of deep CNN. It has numerous advantages. The CNNs possess the capability to integrate the feature extraction and classification process into one learning body. It can also learn to perform feature optimization during the training stage from raw input. Deep CNN are sparsely associated with connected weights and could process many inputs with high computational efficacy. These are also immune to trivial conversions in input data inclusive of translation, skewing, scaling, and distortion. Deep CNNs could also adapt to varied input sizes. Generally, Deep 2D CNN is employed on images, and its overall architecture is shown in Figure 2, which comprises numerous layers, such as batch normalization, maxpooling, dropout, flatten, and dense layers. It is called 2D CNN, with kernel slides with two data dimensions.

Feature extraction from OPG images is performed by Deep 2D CNN based on the below procedure.

1. Input Layer

Features from pre-processed data are declared as an input of Deep 2D CNN and are given by Equation (1).

(1) $$X=[x_{1},x_{2},x_{3},\ldots ,x_{numF}]$$

In Equation (1), represents the feature count per window after computation. To enhance the speed of model’s convergence, min–max normalization is used (as shown in Equation (2)), by which values in the individual data dimension are linearly converted. Then, they are normalized to a range of [0, 1].

(2) $$x=\frac{x-min}{max-min}$$

In Equation (2), represents minimum of individual column, while represents the maximum of the individual column.

2. Convolution layer

The output from the feature map residing on the unit of the convolution layer is given by Equation (3).

(3) $$x_{i}^{l,j}=\sigma (b_{j}+\sum_{a=1}^{m}w_{a}^{j}x_{i+a-1}^{l-1,\;j})$$

where represents the bias for feature map, size of kernel is indicated by, indicates the weight of feature map, represents filter-index, and represents activation function.

3. Maxpooling layer

The pooling layer gives descending aggregation measurements for the adjacent outcome that could minimize the dimension as well as the output sensitivity, accomplishing scale invariant feature maintenance. Maxpooling is the pooling function utilized in this study that divides the convolutional layer’s output features into various partitions and determines the maximum in the individual partition. The output of this layer is given by Equation (4).

(4) $$x_{i}^{l,j}={max}_{pos=1}^{r}(x_{\left(i-1\right)*T_{ps}}^{l-1,j})$$

In Equation (4), indicates pooling stride, and represents the pooling size.

4. Training the model

Deep CNN comprises numerous layers, such as Conv2D, batch normalization, maxpooling 2D, dropout, flatten, and dense layers. Then, input is mapped into the feature space of the hidden layer. Finally, the dense layer integrates varied features of local structure learned from the lower layer for performing final prediction. This study used a single pair of a convolution layer and a maxpooling 2D layer. Subsequently, the 2D data are flattened into 1D data; in this way, the overall neural network is completed with a dense layer and is given by Equation (5).

(5) $$f(x)={argmax}_{cls}(\frac{e^{x^{l-1}w_{j}}}{\sum_{n=1}^{N}e^{x^{l-1}w_{ps}}})$$

In Equation (5), indicates class label, represents features of a sample, indicates layer index, and indicates the class count. Further, forward propagations are processed in accordance with Equations (3) and (5). The information proliferates forward from the input layer by the hidden layer to the output layer and thereby accomplishes the output of the overall network. Moreover, an iteration of forward propagation affords the network error value. Cross-entropy cost function computes the error value as given in Equation (6).

(6) $$L\left(y\right)=-\frac{1}{n}\sum_{x}\left[ylna+\left(1-y\right)\ln\left(1-a\right)\right]$$

In Equation (6), indicates sample, represents total training samples, and indicates actual value, while represents predicted value. The overall algorithm of Deep 2D CNN is given in Algorithm 1.
**Algorithm 1**: Deep 2D CNN1Input: OPG Images2Output: Features3**STEP 1**: Sliding Window Process4**STEP 2**: sef ← Extract Shadow Features5**STEP 3**: Normalize sef using equation (2)6regularization feature data, size = 64 Units–128 Units7repeat:8**STEP 4**: Forward Propagation9cdf ← Convolution2D(sef);10mp ← Max_pooling(cdf);11fc ← Fully_connected(mp);12class label ← relu(fc);13**STEP 5**: Backward Propagation14conduct backward propagation with Adam;15Until wi convergences;// wi: weight16**STEP 6**: Use the trained network to predict the features

Initially, OPG images are taken as input. Then, the sliding window process is performed. Following this, the shadow features are extracted. Then, these features are normalized and regularized using Equation (2). Subsequently, forward and backward propagation is performed until the weight converges. Finally, the trained model is used for predicting features.

#### 3.3.2. Deep 1D CNN: Deep One-Dimensional Convolutional Neural Network

Deep 2D CNN is an altered version of Deep 1D CNN, which has gained interest in recent times. It requires minimum computational requirements and is suited for low-cost and real-time applications. Though mainstream models mostly rely on 2D convolution, the main idea of 1D CNN is almost the same. The main variation is that 2D convolution operates with information of the matrix, wherein 1D convolution operates with 1D vector information and can be expressed by the following:

(7) $$x_{l}=conv1d\left(w_{l-1},x_{l-1}\right)+b_{l}$$

In Equation (7), represents the results, and indicates bias.

Non-linear functions are added to fit the actual function in a better way. So, the overall outcome is indicated as follows:

(8) $$y_{l}=f(x_{l})$$

In Equation (8), represents ReLU activation functions or sigmoid functions.

In CNNs, there exists a significant concept called receptive fields, which represents the region where input data could be viewed by features of the CNN. When (3 × 1) kernel size is utilized, the initial hidden layer can view the three characteristic original data values, and the subsequent hidden layer can view five characteristic original data values. Thus, utilizing two (3 × 1) convolution kernels instead of single convolution kernel having size (5 × 1) could minimize the parameters while confirming that a similar receptive area is attained. In accordance with this idea, the block below was framed using numerous small kernels for extracting features.

#### 3.3.3. Feature Concatenation

After features were extracted from OPG images and patient records, they were concatenated, and these features were fed into train and test split for age estimation. The total number of OPG images was 622, and total number of records was also 622. Out of 622 OPG images, 60 images were extracted, while out of 622 patient records, 15 features were extracted. Upon concatenating, a total of 75 features were obtained.

### 3.4. Regression-MG-RF (Modified Genetic–Random Forest)

**Figure 3:**
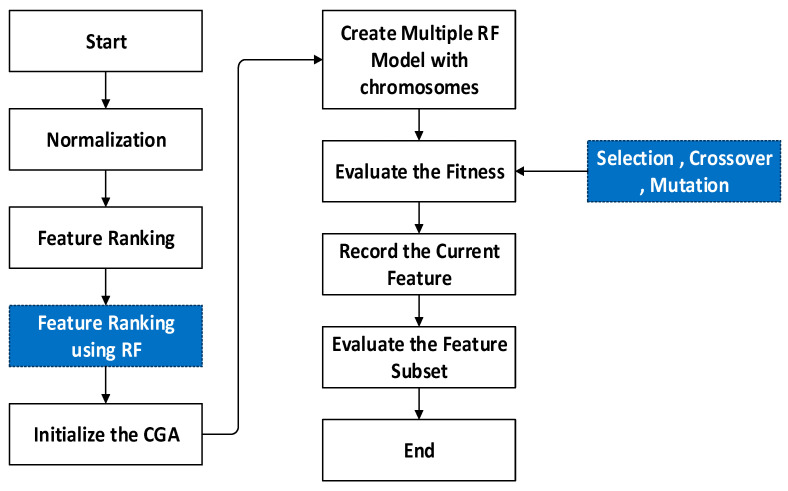
Overall workflow of Modified Genetic–Random Forest.

Generally, GA (Genetic Algorithm) is a general-purpose and optimization search method relying on genetic theory and the natural selection of Darwin in biological systems. In this algorithm, the population individually is called a chromosome, which relates to a resolution for a specific issue. The individual chromosome indicates a combination of DT (Decision Tree), so the length of an individual chromosome indicates total DT, and when DT possesses a value of 1, DT is retained, while value 0 represents that DT is denounced. On the other hand, RF adds additional randomness to model while developing trees. Rather than searching a significant feature during node split, it searches the suitable feature within a random feature subset. This leads to extensive diversity and a better model. Due to such advantages, this study considered GA and RF together as MG-RF to estimate age by the specific, distinct process shown in Figure 3.

Initially, features are normalized, wherein the numeric column values in the dataset are changed to common form without distorting variations in value ranges. This assists in solving the learning challenges of a model. After this, feature ranking is performed using RF for considering important features. Following this, Compact Genetic Algorithm (cGA) is initialized. Subsequently, multiple RF models are created with chromosomes. Then, the fitness value is evaluated using the selection, crossover, and mutation processes of GA. These operations transform the chromosome’s initial population, improving the quality. The selection represents the process that chooses parents that mate and then reunite for developing offspring for the succeeding generation. This is vital for the convergence of GA, based on which it can obtain the best solutions. The selection process uses fitness for controlling chromosome evolution. Maximum fitness affords more chances to select optimal solutions, whereas crossover involves the integration of the genetic material as well as the elite position chosen for the crossover operation. Further, mutation is employed in a chromosome at the individual position. Finally, the fitness value is assessed, and the current feature is recorded. Then, the feature subset is evaluated. For a specific issue, the fitness function improves the chromosome quality as a solution. Hence, it assists in attaining better age estimation. The overall algorithm of GA is shown in Algorithm 2.
**Algorithm 2:** Genetic algorithm1Input: (it, n, GA Parameters)2**STEP 1:** begin3**STEP 2:** Initialize c = 0 and i = 0,4**STEP 3:** Generation: generate random n solutions;5**STEP 4:** Compute Fitness(s) and Generation c;6**STEP 5:** While fitness not reached compute for i iterations do7Generation c + 1 evolve(Generation c);8**STEP 6:** fitness computeFitness (s) and Generation c;9 10 11 12i = i + 1; end return (solution fitness) end

At first, the parameters are initialized. Then, random solutions are generated. Subsequently, fitness and generation are computed. When a suitable fitness value is not found, iterations are performed until the best fitness solution is attained. After finding this value, the best parameters are attained through the use of the obtained best fitness value. The algorithm corresponding to this process is shown in Algorithm 3.
**Algorithm 3:** Modified fitness computation1Input: Dataset(D), Chromosome2Output: MAE of the Random Forests3**STEP 1:** begin4**STEP 2:** Ds—Dataset;5**STEP 3:** Compute kvalues, num_trees, mtry by decoding (Chromosome); 6Dc—decompose the set as (Ds, kvalues);7**STEP 4:** Fitnessmodel—RF fit(Dc,num_trees,mtry);8**STEP 5:** Rank the feature using RF Regressor9 10 11**STEP 6:** MAE—evaluate(model) **STEP 7:** return (MAE); end

Initially, the dataset and chromosome are taken as input. Then, they are computed by decoding. Following this, the sets are decomposed. Subsequently, the fitness model is determined. After this, the features are ranked using an RF regressor. Finally, the MAE of a model is assessed. Lastly, the best parameters are attained from GA, as shown in Table 1.

After extraction of the best parameters, the optimized solution is obtained based on Algorithm 4.
**Algorithm 4:** Optimized Random Forest1Input: minK, maxK, minNTree, maxNTree, treeIncrement, RF best, RF fit2Output: Optimized RF3**STEP 1:** begin4**STEP 2:** Compute computeFitness(s) and Generation c;5**STEP 3:** Evaluate fitness and return fitness,6**STEP 4:** MAE (Fit RF best)7**STEP 5:** Fit RF best = Optimized RF8**STEP 6:** Optimized RF(D) = solution
9end

At first, the input is considered. Then, the fitness value is computed. Following this, the fitness value is evaluated and returned. Subsequently, the fitness value is assessed to determine the solution. After this, the hyperparameters for RF are attained, as shown in Table 2.

The main stages involved in the outcome of the optimized RF are summarized below.

**Step 1: Modified genetic RF**

MG-RF is trained, and the fitness value is evaluated with GA. In this phase, the classes obtained are optimized in accordance with GA, and this attains the input for RF that calculates the fitness of individual trees in a forest. Iteration continues until the optimized trees are determined.

**Step 2: Fitness calculation**

In this stage, the overall fitness of trees is assessed based on the values attained through decoding the chromosomes. The decomposed class obtained from phase 2 is assessed for its fitness value with ranked features corresponding to MAE score.

**Step 3: Optimized RF**

The optimized RF is attained from integration of GA (with the assistance of selection, crossover, and mutation processes) and multiple class decompositions.

**Step 4: Termination**

The operation is terminated after obtaining the optimal RF.

**Step 5: Outcome**

This stage involves predicted data for estimating age.

Hyperparameter tuning exerts a substantial influence on the performance of deep learning DL models. The integration of the GA with the RF algorithm not only enhances the predictive accuracy but also improves interpretability. The GA method was used to adjust the hyperparameters of the Random Forest to improve its regression accuracy.

Additionally, GA was designed to optimize dental age estimation, and the population size was set at 20 individuals, allowing for a diverse representation of potential solutions. The algorithm was run for 50 generations, providing ample opportunity for the population to evolve and improve over time. The fitness function utilized was the MSE, calculated on validation data, ensuring that the solutions were evaluated based on their predictive accuracy. To select individuals for reproduction, tournament selection was employed, which enhanced the chances of selecting high-quality solutions. The crossover rate was set at 0.8, thereby facilitating a robust exchange of genetic material between parent solutions, while a mutation rate of 0.1 introduced variability and aided in the prevention of premature convergence. This structured approach was designed to effectively navigate the solution space and achieve optimal results.

The following Table 3 describes the number of layers and size of the kernel utilized in the Deep 2D CNN and Deep 1D CNN architecture. Accordingly, the Deep 2D CNN analyses the OPG images with four convolutional layers, with progressively increasing filter counts, followed by maxpooling and fully connected layers. The Deep 1D CNN processes patient records with three convolutional layers, each generating a 128-unit feature vector as output. The output features from each network are then combined in a fusion layer. Following that, the outcome is fed into an MG-RF regressor to enhance the predictive accuracy.

## 4. Results

**Figure 4:**
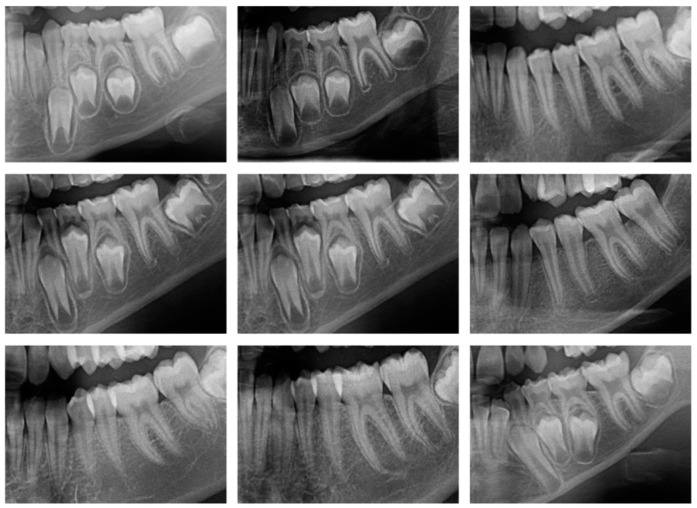
Sample OPGs from the dataset.

This study utilized the OPG images and patient records within the dataset. A real-time dataset was considered for this research work. Some sample OPG images from the dataset are shown in Figure 4. Out of 275 males, the average age was 10.94, while out of 346 females, the average age was 11.1.

### 4.1. Performance Metrics

The metrics considered for analysis of the proposed methods are discussed in this section.

#### 4.1.1. SD (Standard Deviation)

Standard deviation represents the computation of dispersion or variation on a particular set of values. Minimum standard deviation denotes that values incline to be close to the mean of set, whereas maximum standard deviation denotes that values are extended on a wide range, as given by Equation (9).

(9) $$\sigma =\frac{\sqrt{\sum {\left(x_{i}-\mu \right)}^{2}}}{N}$$

In Equation (9), σ indicates standard deviation, N represents the population size, μ indicates mean of the population, and $x_{i}$ denotes the individual value from the population.

#### 4.1.2. MAE (Mean Absolute Error)

This is an assessment metric of a model utilized with regression models. The MAE of a model in terms of the test set could be indicated as the mean of the absolute values corresponding to the individual errors of prediction upon cases of the test set, afforded by Equation (10).

(10) $$MAE\left(Mean\;Absolute\;Error\right)=\frac{\sum_{i=1}^{n}\left|predicted\;value-actual\;value\right|}{n}$$

In Equation (10), n represents the overall data points.

#### 4.1.3. MSE (Mean Square Error)

This explores the closeness of a set of regression lines and points, as given in Equation (11).

(11) $$MSE=\frac{1}{n}\sum_{i=1}^{n}\left(observed\;values-predicted\;values\right)\;$$

#### 4.1.4. R^2^ (Coefficient of Determination)

The coefficient of determination refers to computation of the goodness of model fit. In regression, R^2^ indicates the statistical computation of the degree to which regression predictions determine the actual data points. When R^2^ = 1, it means that the regression estimations perfectly fit the corresponding data, as given by Equation (12).

(12) $$R^{2}=1-\frac{Sum(squares\;of\;the\;residuals)}{Total\;sum\;of\;the\;squares}$$

#### 4.1.5. RMSE (Root Mean Square Error)

The RMSE is the ideal accuracy calculation that only compares model configurations or prediction errors of diverse models for specific variables. It is given by Equation (13).

(13) $$RMSE=\frac{\sqrt{\sum_{i=1}^{N}{\left(actual\;time\;series\;observation-predicted\;time\;series\;observation\right)}^{2}}}{N}$$

In Equation (13), N represents overall non-missing data-points, and i indicates the variable.

### 4.2. Performance Analysis

**Figure 5:**
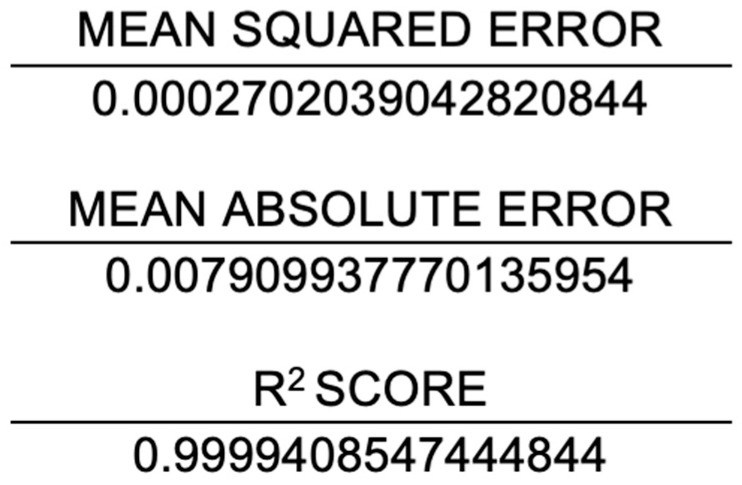
Analysis of the proposed system with respect to metrics. In statistics, the mean squared error of an estimator (of a procedure for estimating an unobserved quantity) measures the average of the squares of the errors—that is, the average squared difference between the estimated values and the true value. Mean absolute error is a measure of errors between paired observations expressing the same phenomenon. R-squared (R^2^) score is defined as a number that tells you how well the independent variable(s) in a statistical model explains the variation in the dependent variable.

**Figure 6:**
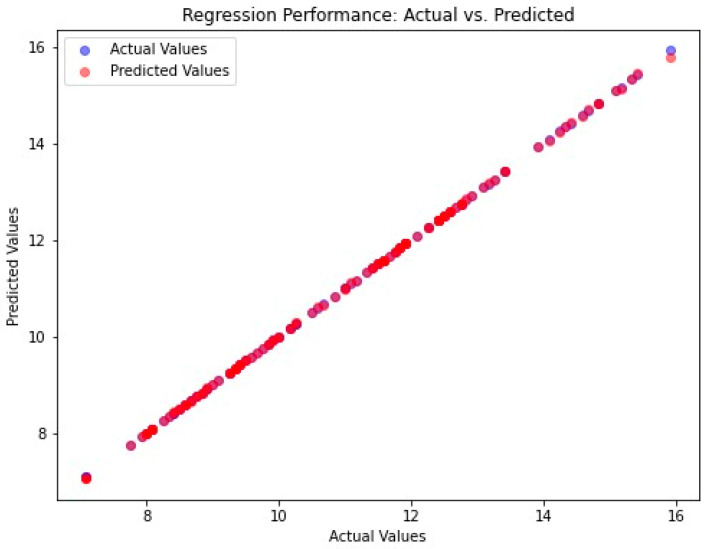
Actual vs. predicted values for proposed model.

**Figure 7:**
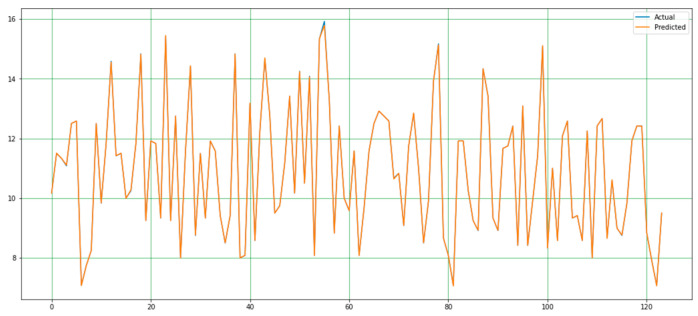
Plot showing variation degree between the actual and predicted values.

In this study, the performance of the proposed system was evaluated based on the mean MSE, MAE, RMSE, and R^2^ values calculated during the implementation of the code. The efficacy of a system is gauged by its maximum prediction rate and minimum error rate. In this regard, the maximum R^2^ score serves as a measure of the proposed system’s effectiveness. In this study, the MSE value of the proposed system was 0.00027, the MAE value was 0.0079, the RMSE value was 0.0888, and the R^2^ score was 0.999. This is shown in Figure 5. A comparative analysis of the predicted and actual values is presented in graphical form in Figure 6 and Figure 7.

Figure 6 presents a scatter plot, which is a graphical representation of the relationship between actual and predicted values in regression analysis, thereby indicating the performance of the model. A model that functions optimally aligns points along the diagonal, where the predicted value is equivalent to the actual value. This is referred to as “Regression Performance of Actual vs. Predicted”. The figure includes an x-axis, which represents actual values, and a y-axis, which represents predicted values. Blue points denote actual values, while red points represent predicted values. The substantial overlap of red points with the blue points, indicating strong model accuracy, is evident in the figure. This overlap is further demonstrated by the majority of red points aligning with the diagonal line y = x without bias, suggesting a high degree of model accuracy.

## 5. Discussion

In the field of forensic science, the determination of an individual’s age is of paramount importance. Consequently, the methodology employed must be both reliable and accurate. This indicates that the degree of accuracy should be high, and the mean discrepancy between dental age and chronological age should be as minimal as feasible [66].

An existing study [78] employed a two-stage approach with the efficientNetV2 model for automatic dental age estimation. It obtained outcomes with MAE 0.274, MSE 0.261, and expected value 0.396. Similarly, another study [81] implemented the estimation of age using an ML approach only on OPG images. The model performance was analyzed with InceptionV4 and achieved MAE metrics of 3.1 and an R^2^ value of 95.5%. Another existing study [79] implemented the DentAge model based on a DL approach for predicting chorological age. It used a transfer learning strategy for training the model with gradient descent features. It attained an MAE of 3.12 years and MAE of 1.94 for the age group between 10 and 20.

However, the current study employed DL models such as Deep 2D CNN for spatial features from OPG images and Deep 1D CNN for extracting sequential data from patient records. Moreover, an MG-RF regressor was employed to enhance the predictive accuracy and obtained a low MAE of 0.0079, RMSE of 0.0888, and R^2^ of 0.999. Hence, the proposed model indicates efficiency in automatically predicting dental age estimation and is suitable for the forensic field.

The age estimation methods that have been documented in the existing literature are based on the evaluation of specific indicators that assess the stage of dental development that the individual has reached. The earliest known studies on this subject are dated to the 19th century [17]. One of the earliest methods based on dental indicators is the Schour and Massler method, which was developed in 1941. This method involves the morphological evaluation of tooth development through the use of diagrams that illustrate the expected developmental stages of deciduous and permanent teeth [82]. Subsequently, Nolla et al. [83] developed a ten-stage chart for age estimation, which was considered an important milestone in the field of age estimation, prompting the rapid development of new methods [11,17,83,84,85]. Currently, in the literature, the most commonly used methods for age estimation in children are reported to be the Willems and Demirjian methods [66].

Demirjian et al. [84] developed a method that can be used as a universal tool for assessing dental maturity and estimating dental age in children. Demirjian’s method is the first in the literature to provide visualization of the stages of tooth development, descriptive criteria, radiographic examples of each stage, and selection rules for decision making at borderline stages. In the Willems method, the stages of tooth development described in the Demirjian method are used to estimate tooth age by means of maturity tables that provide the age in years directly [11,84,85].

The classical methods that have been introduced in the literature have been developed through the analysis of large datasets comprising a large number of participants. However, these methods have several shortcomings, including the fact that results are often population-specific and rely on time-consuming manual procedures that are susceptible to observer subjectivity [16,86].

A further limitation of classical manual radiologic tooth age estimation techniques is that they lack the requisite number of stages to enable the closest possible monitoring of the growth process. Another disadvantage is the difficulty in selecting a method that allows researchers to distinguish teeth that are not sufficiently differentiated in terms of developmental stage [16].

The objective of the research was to estimate the age of patients from a real-time dataset comprising patient records and OPG images using AI-based methodologies. This study employed Deep CNN with 1D and 2D architectures for feature extraction and an MG-RF method for estimating age. The proposed system was evaluated by examining the discrepancies between the actual and predicted values. The results demonstrated a strong correlation between the two variables. Furthermore, the performance of this system was evaluated in comparison to that of the conventional system in terms of MAE, RMSE, MSE, SD, and R^2^ score. The analytical outcomes revealed that the proposed system outperformed the conventional system, exhibiting an SD rate of 0.0004, an MAE rate of 0.0079, an MSE rate of 0.00027, an RMSE value of 0.0888, and an R^2^ value of 0.9999.

The performance of the reference methods and machine learning algorithms utilized in the study conducted by Galibourg et al. [66] was evaluated with analogous metrics to those employed in our study, namely R^2^, MAE, RMSE, and SD. The researchers reported that age estimation with machine learning methods demonstrated superior performance compared to manual methods based on radiographic tooth staging from childhood to early adulthood. The results of the study are presented in Table 4, along with a comparison to the findings of our study.

The study of Galibourg et al. [66] was carried out with a methodology based entirely on machine learning. The Demirjian and Willems methods were used as the explanatory system in the training of the machine learning system. These methods focus on the left seven mandibular permanent teeth for age estimation.

Our study was carried out with the aim of performing age estimation on OPG sections containing the left seven mandibular permanent teeth in a fully automatic manner without any explanatory system. From the radiographs and patient records that constitute the dataset of our study, feature extraction was performed using 1D-DCNN and 2D-DCNN architecture from deep learning methods. In the regression step, RF and GA methods were modified and combined, and age estimation was performed.

When our study is compared with the study of Galibourg et al. [66], in terms of performance, it is observed that our study has a superior performance than the manual predictions and all machine learning approaches evaluated in the related study. In addition, it was found that the MAE value decreased significantly with the system used in our study. It is thought that the reason for the decrease in the MAE value may be due to the use of deep learning algorithms in the feature extraction step of our study due to the perception by deep learning algorithms of various age-related indicators that cannot be detected by the human eye, and thus, more information is transferred to the MG-RF algorithm. In the MG-RF step, the RF algorithm reduces the similarity between individual trees as a methodology. For this reason, the robustness of the final model was increased by the selection of the point of departure from a random subset of the input features at each step in the tree-building process. In addition, in our study, it is thought that the integration of four different approaches into the system together with the use of machine learning techniques following deep learning processes resulted in a significant decrease in the error rate and a significant increase in the performance of the system.

In our study, age estimation was performed completely automatically without using any explanatory reference system. The fact that our method eliminates the disadvantages of exposure to human interpretation and the subjectivity of human observers by automating the age estimation task can be argued as a distinct advantage of our study. In addition, the fact that no explanatory system is used and that it is an automatic method makes our study easy to use, fast, and reproducible.

Tao et al. [67] proposed a machine learning-based approach to improve the accuracy of tooth age estimation in 2020 using a dataset of 1636 OPGs of 787 male and 849 female individuals aged 11 to 19 years. In the study, tooth age estimation was considered as a regression problem.

In the methodology of the study, manual measurements were first performed using the Demirjian method and the Willems method. The attributes were determined by entering the real ages of the patients into the system. Then, the MLP algorithm, which is a feed-forward artificial neural network from machine learning approaches, was trained with these features, and experiments were conducted. The performance of the proposed system was evaluated using MAE, MSE, and RMSE metrics. It was reported that the proposed system outperformed the reference manual methods in terms of all performance metrics [67]. The findings of the study and the findings of our study are presented in Table 5.

The findings of our study, as indicated by the RMSE, MSE, and MAE metrics, demonstrate significantly enhanced performance in comparison to the results obtained for both female and male groups in the study conducted by Tao et al. [67]. This discrepancy may be attributed to the fact that the group under examination in our study comprises younger individuals (6–15 years old) as well as the variations in the age distribution of these individuals. Given that age indicators of growth and development decline with age, it is a well-established fact that studies in the field of age estimation obtain more accurate results with younger study populations [17].

In our study, it is thought that the combination of deep learning and machine learning methods significantly enhanced the performance. The use of deep learning techniques allows for the establishment of connections that are not discernible to the human eye, thereby enabling the inclusion of age indicators that cannot be calculated manually in the system under study. It can be proposed that this may be the source of the observed improvement in the system’s performance. Furthermore, we employed an automated system that is not contingent on any explanatory framework. Therefore, our method has the advantages of being fast, repeatable, and less susceptible to human interpretation.

In their 2021 study, Shen et al. [68] employed a series of machine learning systems to estimate age. The dataset utilized in the study comprised 748 OPGs of 356 female and 392 male individuals between the ages of 5 and 13. The study employed a methodology based on random forest (RF), support vector machines (SVM), and linear logistic regression (LR). The machine learning models were trained with the manually realized Cameriere method as an explanatory system and gender information. The target value was set to the subject’s chronological age. The accuracy of the proposed systems for estimating age was evaluated based on the following metrics: R², ME, RMSE, MSE, and MAE. The results were then compared with those obtained using the European and Chinese formulas of the Cameriere method, which were employed in the training of the system. The findings of the study and the findings of our study are shown in Table 6 [68].

The performance of the systems proposed in the study is comparable to that observed in our own study in terms of the R² value. It is, however, noteworthy that the error rates of the systems proposed by Shen et al. are significantly higher than those observed in our study with respect to the other performance metrics evaluated. It is thought that this discrepancy may be attributed to the utilization of deep convolutional neural networks in the feature extraction phase of the present study, the incorporation of certain age indicators that cannot be discerned by the human eye, and the combination of numerous techniques.

In 2017, Čular et al. [69] conducted a study utilizing OPGs of 203 individuals between the ages of 10 and 25. In this study, the researchers proposed a semi-automated system based on deep learning techniques to estimate tooth age by examining the mandibular right third molar on OPGs. The researchers employed two statistical computer vision models, namely the Active Shape Model (ASM) and the Active Appearance Model (AAM), which have been extensively utilized in face recognition, gender estimation, and medical image interpretation and feature extraction in previous studies, to extract the features describing the right mandibular wisdom teeth selected for this investigation. In the training set, the images were manually segmented. In the study, the extracted features were presented as input to an artificial neural network, specifically the Radial Basis Network, and age estimation was performed as the output. The findings of the age estimation performance of the study were evaluated using the mean absolute error (MAE) in years. The findings of that study indicated that the system demonstrated superior performance when AAM feature extraction was employed. Although AGM was reported to perform better in this study, it was reported that the age estimation performance of the system was adversely affected when AAM and ASM were applied together [69].

The researchers noted that the MAE value of less than 3 years represents a promising preliminary result. Should the prediction error be reduced in future studies, the system proposed in their study may prove a viable option for use within the scope of forensic sciences. Furthermore, the researchers indicated that the system proposed in their study offers two key advantages: minimal user input and the ability to function without the input of an experienced dentist [69]. The findings of the study and the findings of our study are shown in Table 7.

A direct comparison between our study and that of Čular et al. [69] is not feasible due to differences in methodology and age group. As there is a greater number of age indicators of growth and development in younger individuals within the existing literature, the MAE value is typically reported to be lower in studies involving this age group. The present study focuses on individuals between the ages of 6 and 15. Accordingly, the lower MAE value observed in our study relative to that reported by Čular et al. is an anticipated outcome.

In contrast to the present study, the fact that our study does not entail a manual step such as segmentation over radiographs while estimating age can be demonstrated as an advantage of the system utilized in our study. Furthermore, as our study employed OPG sections encompassing the left seven mandibular permanent teeth, the third molars, which are the most common congenitally missing teeth, were not required, thus conferring another advantage to our study.

In 2021, Wallraff et al. [71] conducted a study on automatic age estimation to reduce the estimation error, which is a disadvantage of traditional manual age estimation methods. In this study, the researchers proposed a deep learning system based on supervised regression to perform age estimation. The study was conducted on 14,000 OPGs of individuals between the ages of 11 and 20. The system proposed in the study uses raw OPGs as input. Images do not need to be pre-processed and cropped. The researchers reported that the focus of this study was individuals 11–20 years of age, as teeth develop in a predictable pattern during the first two decades of life. Radiographs in the dataset that were affected by various external factors, such as low-contrast images, diseases, and jaw malpositions, were not excluded. In this study, age estimation was considered as a regression problem, and the ResNet18 algorithm was used as the network architecture in the proposed system. The MAE of the proposed system was reported to be 1.08, with an SD of +1.41 and an error rate of 17.52%. The researchers stated that the dataset in their study provided comprehensive data for the age range of 11–20 years and that individuals aged 0–20 years should be included in the proposed system in the future [71].

As indicated in the literature, age-related indicators tend to decline as growth and development progress, resulting in lower error rates in studies that examine younger age groups. In the study conducted by Wallraff et al. [71], individuals below the age of 11 were excluded from the analysis. A comparison of our study with that of Wallraff et al. revealed a significantly lower MAE value for our study. It is postulated that this finding is attributable to the fact that our study focused on younger individuals.

In their 2020 study, Vila-Blanco et al. proposed a method based on deep learning algorithms for fully automatic age estimation from OPGs. This method was designed to overcome the limitations of traditional methods, which are affected by observer subjectivity and time-consuming manual operations. The study was conducted on OPGs of 2289 individuals from a Spanish population with an age range of 4.5 to 89 years. Only OPGs of the requisite quality were included in the study. In contrast to other studies in the literature, this study did not exclude OPGs with any of the following: orthodontic brackets or appliances, dentures, implants, restorative materials, fillings, endodontic treatment, foreign bodies such as caries, missing teeth, residual tooth roots, or earrings and external elements such as distorted or blurred images. The images in question were identified as defective within the system. In this study, convolutional neural networks from deep learning algorithms were employed as the methodology. Two distinct network architectures, namely DANet and DASNet, were devised for the purposes of this study. These architectures were designed and trained specifically for the purpose of age estimation in the context of this study [16].

The results of the study indicate a strong correlation between the DANet and DASNet systems and age. The coefficient of determination was R^2^ = 0.87 for DANet and R^2^ = 0.90 for DASNet. In the assessment of the data across all age groups, MAE was found to be 2.84 years. As the age of the individuals decreased, the MAE value was found to be 0.78 years for DANet and 0.75 years for DASNet in the group of individuals younger than 15 years.

In contrast to the methodology employed by Vila-Blanco et al. [16], our study utilized an OPG section encompassing seven left mandibular permanent teeth as the input data for the system. In the study conducted by Vila-Blanco et al. [16], the raw OPGs were utilized as the input data, and no exclusion criteria were employed. However, radiographs with inadequate acquisition quality were designated as defective and submitted to the system. The present study was conducted using high-quality radiographs of individuals without any dental or bony pathology. It is believed that this contributed to the success of the system used in our study.

With regard to the age group under examination, our study encompasses a much younger population than that considered by Vila-Blanco et al. Given that developmental age indicators are more prevalent in younger age groups, it may be posited that the superior performance of the Vila-Blanco et al. study can be attributed to the findings of our study. Moreover, the integration of deep learning and machine learning methodologies is believed to be a contributing factor to the enhanced performance of the system in our study.

A series of ablation experiments was conducted to assess the contribution of the components utilized in our study to the overall model performance and to enhance interpretability. Upon evaluating the method proposed in the current study, it was observed that the highest performance was achieved with a low mean absolute error (MAE) of 0.0079 and a high R^2^ of 0.999. These findings underscore the efficacy of the proposed method and underscore the critical role of feature fusion. Table 8 provides a comprehensive overview of the performance of various experimental configurations, facilitating a nuanced evaluation of the proposed work using performance metrics.

## 6. Conclusions

The application of AI methodologies has the potential to significantly reduce the time required for the resolution of complex problems. Moreover, these systems provide consistent and reproducible high-performance results.

The proposed systems with deep learning algorithms show exceptional performance due to their capacity to distinguish features that are imperceptible to the human eye and to discern the relationships between these features. However, the underlying logic behind the outstanding performance of these systems has not yet been fully elucidated, which constitutes a disadvantage for the interpretability of these systems. As in other studies in this area, interpretability is a limitation in our study. In order to address this limitation, a series of ablation experiments was conducted with the objective of enhancing interpretability. To increase interpretability and enhance the efficiency and reliability of real-world applications, in future work, we will use methodologies such as Class Activation Maps (CAM) to gain real-time insights into model predictions, interactive dashboards integrating SHAP and LIME visualizations, and Deep LIFT and Integrated Gradients to provide more understandable explanations of deep model predictions.

The present study focuses on individuals aged 6–15 years. In the field of forensic sciences, accurate age estimation is paramount, particularly in cases involving legal issues related to age and identification of elderly individuals. This represents a limitation of the present study. In subsequent studies, efforts will be directed towards the development of automatic age estimation methods for study populations that include individuals belonging to older age groups.

In conclusion, contemporary AI-based techniques in the field of forensic sciences have reached a point where they are capable of providing substantial assistance to human analysts. It is of significant importance to conduct future studies that will test the potential of these techniques to supplant human analysts and to elucidate the reasons behind their performance.

## Figures and Tables

**Table 1:** Best parameters from GA.

Best Parameters from Genetic Algorithm	
max_depth’	[10]
max_features’	[sqrt]
min_samples_leaf’	[4]
min_samples_split’	[10]
n_estimators’	[600]

**Table 2:** Hyperparameters for RF.

Hyperparameters For RF	
‘max_depth’	[10, 20, 30, 40, 50, 60, 70, 80, 90, 100, None]
‘max_features’	[‘auto’, ‘sqrt’]
‘min_samples_leaf’	[1, 2, 4]
‘min_samples_split’	[2, 5, 10]
‘n_estimators’	[200, 400, 600, 800, 1000, 1200, 1400, 1600, 1800, 2000]

**Table 3:** Components in Deep CNN architecture.

Model	Component	Details
Deep 2D CNN	Input Layer	Input size: (224, 224, 3) (Resized OPG Images)
	Convolutional Layers	Conv1: 32 filters, kernel size (3 × 3), ReLU Conv2: 64 filters, kernel size (3 × 3), ReLU Conv3: 128 filters, kernel size (3 × 3), ReLU Conv4: 256 filters, kernel size (3 × 3), ReLU
	Pooling Layers	Maxpooling after each convolutional block, pool size (2 × 2)
	Fully Connected Layer	Dense layer: 512 units, ReLU activation
	Output Layer	Dense layer: 128 units (feature vector), Linear activation
Deep 1D CNN	Input Layer	Input size: Variable (Patient Records)
	Convolutional Layers	Conv1: 16 filters, kernel size (5), ReLU Conv2: 32 filters, kernel size (3), ReLU Conv3: 64 filters, kernel size (3), ReLU
	Pooling Layers	Global Maxpooling layer after final convolutional layer
	Fully Connected Layer	Dense layer: 256 units, ReLU activation
	Output Layer	Dense layer: 128 units (feature vector), Linear activation
Feature Fusion	Concatenation Layer	Combines 128-unit outputs from both Deep 2D CNN and Deep 1D CNN
Modified Genetic-RF	Feature Input	256 features (128 from Deep 2D CNN + 128 from Deep 1D CNN)
	Regressor	Random Forest with Genetic Algorithm optimization: - Number of Trees: 100 Maximum Depth: Optimized by Genetic Algorithm- Split Criterion: Mean Squared Error

**Table 4:** Comparison of the findings of Galibourg et al.’s [66] study with the findings of our study.

Methods	SD	MAE	MSE	RMSE	R^2^
Demirjian	−0.705	1.108	1.981	1.406	0.816
Willems	−0.220	0.928	1.418	1.190	0.868
BRR	−0.002	0.812	1.030	1.014	0.904
SVM	0.016	0.729	0.901	0.949	0.916
DT	−0.012	0.758	0.973	0.985	0.910
RF	−0.007	0.731	0.885	0.940	0.918
KNN	0.009	0.738	0.921	0.959	0.915
MLP	−0.041	0.742	0.907	0.952	0.916
POLYREG	−0.008	0.735	0.913	0.955	0.915
ADAB	−0.025	0.796	1.001	1.000	0.907
STACK	−0.013	0.733	0.904	0.950	0.916
VOTE	0.068	0.770	0.995	0.984	0.908
The proposed method	0.0004	0.0079	0.00027	0.0888	0.9999

**Table 5:** Comparison of the findings of Tao et al.’s study [67] with the findings of our study.

Male	RMSE	MSE	MAE	Female	RMSE	MSE	MAE
Demirjian	1.596	2.548	1.307	Demirjian	1.677	2.812	1.364
Willems	1.602	2.556	1.291	Willems	1.788	3.196	1.407
MLP	1.332	1.775	0.990	MLP	1.617	2.616	1.261
The proposed method	0.8888	0.00027	0.0079	The proposed method	0.8888	0.00027	0.0079

**Table 6:** Comparison of the findings of Shen et al.’s [68] study with the findings of our study.

Methods	MAE	MSE	RMSE	R^2^
LR	0.553	0.488	0.698	0.909
SVM	0.489	0.392	0.625	0.925
RF	0.495	0.389	0.623	0.928
Cameriere Method (European Formula)	0.846	0.755	0.869	-
Cameriere Method (Chinese Formula)	0.812	0.89	0.943	-
The proposed method	0.0079	0.0002	0.0888	0.9999

**Table 7:** Comparison of the findings of Čular et al.’s [69] study with the findings of our study.

	MAE	SD
AAM	2.481	2.148
AGM	2.283	2.168
The proposed method	0.0079	0.0004

**Table 8:** Ablation experiment result.

Experiment Configuration	MAE	MSE	RMSE	R²
Proposed Method (Full)	0.0079	0.0002	0.0888	0.9999
Without Deep 2D CNN	0.0125	0.0008	0.1414	0.9985
Without Deep 1D CNN	0.0113	0.0006	0.1225	0.9989
Without Feature Concatenation	0.0150	0.0012	0.1732	0.9978
Using Only Deep 2D CNN	0.0132	0.0009	0.1500	0.9982
Using Only Deep 1D CNN	0.0148	0.0011	0.1667	0.9979

## Data Availability

Data are available from the corresponding author upon reasonable request.
